# Rising floor and dropping ceiling: organ heterogeneity in response to cold acclimation of the largest extant amphibian

**DOI:** 10.1098/rspb.2022.1394

**Published:** 2022-10-12

**Authors:** Wei Zhu, Chunlin Zhao, Tian Zhao, Liming Chang, Qiheng Chen, Jiongyu Liu, Cheng Li, Feng Xie, Jianping Jiang

**Affiliations:** ^1^ CAS Key Laboratory of Mountain Ecological Restoration and Bioresource Utilization & Ecological Restoration Biodiversity Conservation Key Laboratory of Sichuan Province, Chendgu Institute of Biology, Chinese Academy of Sciences, Chengdu 610041, People's Republic of China; ^2^ University of Chinese Academy of Sciences, Beijing 100049, People's Republic of China

**Keywords:** cardiac function, cold adaptation, glycolysis, metabolic depression, metabolomics, thermal compensation

## Abstract

Low temperature imposes strong selective pressure on ectotherms. To maximize their overall fitness under cold conditions, the ectotherms may either try to maintain their physiological activities through metabolic compensation or enter into metabolic depression; however, some species adopt both strategies to cope with different degrees of cold. Nevertheless, how these two seemingly opposite strategies are coordinated has rarely been elucidated. Here, we investigated the molecular strategy underlying the cold acclimation of *Andrias davidianus*, the largest extant amphibian, using multi-organ metabolomics and transcriptomics. The results showed remarkable organ heterogeneity in response to cold. While most organs showed transcriptional upregulation of metabolic processes, the heart exhibited downregulation. This heterogeneity explained the adaptive reorganization in resource allocation, which compensates for metabolic maintenance by compromising growth. Importantly, the cardiac function might constitute a ‘ceiling’ to constrain the space for compensation, especially under colder conditions. Additionally, the opposite transcriptional regulation of oxidative phosphorylation and other pathways might also shape the overall metabolic capacity under cold conditions. The heterogeneity in cold responses may have directed a shift in cold adaptive strategy from compensation to depression with a drop in temperature. These results provide a novel insight into the regulatory mechanisms underlying cold survival strategies of ectotherms.

## 1. Introduction

Low temperature imposes strong selective pressure on ectotherms [1]. It depresses the rate of basic metabolic processes and reduces activities, such as respiration, locomotion, feeding and development, especially when temperatures approach 0°C [2]. These physiological outcomes ultimately exert a marked influence on distribution range and population dynamics [3–5]. Moreover, seasonal cooling is always accompanied with other harsh environmental conditions, including reductions in food, water and even oxygen availability [6]. To maximize their overall fitness, ectothermic animals exhibit a wide range of responses to enable them to cope with ambient temperature variations [7–9].

Many animals have evolved strategies to remodel their physiological processes to compensate for thermodynamic effects (i.e. thermal acclimation or compensation) [9–11], and the thermal acclimation capacity of a species is considered a determinant of its sensitivity to climate change [12,13]. Cold acclimation occurs at the whole animal, organ, organelle and molecular levels to upregulate metabolic activities [14–16]. It is a type of phenotypic plasticity that can alter thermal tolerance limits (e.g. reducing the lower thermal limits for locomotive activity) and optimize physiological performance (e.g. encouraging respiration rate) under thermal stress [17–19]. It allows animals to maintain their physiological activities (e.g. locomotive and feeding activities) at lower temperatures and thus improves the capacity for environmental exploitation [20,21]. Although thermal acclimation can maintain ectotherms in a relatively stable physiological and metabolic state and extend the thermal windows of some physiological activities, it may not be always beneficial if the resource consumed to maintain this state exceeds the acquired amount. Thermal compensation is not likely to offset the thermodynamic effect on physiological activities perfectly, especially when the temperature drops to a relatively low level [14]. When animals enter into a physiologically inactive state, maintaining low metabolic activities should be in favour of long-term survival. Many ectotherms, especially the terrestrial ones, enter into a regulatory low metabolic state to survive winter [22,23], which is called metabolic depression [24,25]. Metabolic depression, in contrast to compensation, is an active downregulation, in addition to the passive thermodynamic effect on energy turnover. Interestingly, some temperate species likely adopt both strategies to cope with the varying environmental temperatures [14,26]. However, how these two seemingly opposite cold adaptive strategies are coordinated in ectotherms to maximize their overall fitness has rarely been investigated.

Despite the great progress in revealing the molecular mechanisms underlying cold adaptative strategies [8,9,27,28], some basic and important questions have not been fully elucidated, i.e. whether there is heterogeneity in thermal responsiveness (e.g. different adaptive strategies) at the organ and molecular levels, and how different organs or cellular processes are coordinated to achieve new homeostasis under cold conditions. These questions are not only implicated in illuminating the molecular mechanisms of environmental adaptation, but also lead the way in revealing the determinants of thermal tolerance in animals. Studies on whole-organ enzyme activities in eastern newts indicate that organ heterogeneity in thermal responses may be a widespread phenomenon [16,29]. The thermal sensitivity of the cardiac system and its thermal acclimation plasticity have become important indexes to evaluate the thermal tolerance of the whole animal [30,31], as the cardiorespiratory system is considered to set the thermal limits of animals at the organismal level [32]. A systematic understanding of organ heterogeneity in thermal susceptibility at the molecular level can be a convictive test for this hypothesis. Moreover, these insights may also shed some light on the coordination between the different cold adaptive strategies.

The Chinese giant salamander (Cryptobranchidae: *Andrias davidianus*) is the largest extant amphibian species, reaching a maximum length of 200 cm [33]. Ancestors of the Cryptobranchidae diverged from other amphibians over 170 million years ago during the Jurassic Period [34]. This makes giant salamanders one of the oldest families on the amphibian tree of life. Thus, *A. davidianus* is renowned as a living fossil, with great significance in physiological and evolutionary studies. *A. davidianus* populations were once widely distributed in central and southern China, and thus different geographical populations likely experienced variable thermal regimes. For example, the monthly mean temperature of a natural *A. davidianus* habitat in the Henan province varies from 1.5°C in January to 25–26°C in July, and these animals cluster and hibernate in caves to survive the winter [35,36]. Laboratory studies have revealed that *A. davidianus* larvae are sensitive to temperature variations [37,38]. Their optimal growth temperature in water ranges from is 15 to 21°C [39]. Our previous studies suggested that *A. davidianus* larvae acclimated to 7°C showed superior cold tolerance and upregulated their metabolic capacity in comparison to their 15°C counterparts [18]. This is suggestive of cold acclimation in this species. Here, the underlying molecular basis was investigated by comparing the thermal physiology, organ-specific (i.e. heart, brain, liver, gill, skin, limb and tail) metabolome and transcriptome between larvae acclimated to 15°C and 7°C. Our main goal is to probe whether these organs respond to cold environment differently at the metabolic level, and how the cellular processes are reorganized to achieve cold acclimation in *A. davidianus* larvae. Given that temperatures lower than 3°C can induce hibernation in these animals [40,41], it is also crucial to understand whether some cold responses at 7°C are in favour of the survival strategy at lower temperatures. Based on these insights, we would like to propose some new thoughts on the switch between physiologically ‘active’ and ‘inactive’ ectotherms facing cold stress.

## 2. Materials and methods

### (a) Animal culture and thermal acclimation

Larvae of the giant salamander from the same clutch were collected from an artificial farm (102^o^10′05″ E, 29^o^52′36″ N) located in Hongya County, Sichuan province in China, where the larvae were maintained at 15 ± 1.14°C (see the details in electronic supplementary material, figure S1). In the laboratory, these larvae were fed daily with red worms (larvae of *Chironomus* sp.). The photoperiod was maintained at 12L : 12D. The animal use protocol in this study was reviewed and approved by the Animal Ethical and Welfare Committee of Chengdu Institute of Biology, Chinese Academy of Science, China.

Thermal acclimation was conducted in 2018 and 2019. The larvae were randomly divided into two groups and acclimated to 15 ± 0.5°C (empirical optimum) and 7 ± 0.5°C (cold, gradually decreased from 15 to 7°C within 2 h). For acclimation in 2018, each group, comprising 35 individuals (approx. 100 days after hatching (d.a.h.); collected on 8 February 2018), was raised in two plastic containers (*n* = 17–18). For the 2019 acclimation, each temperature group, comprising 60 individuals (approx. 140 d.a.h.; collected on 23 January 2019), was raised in three plastic containers (*n* = 20). The size of the plastic containers was 29 × 20 × 9.7 cm, and each container was filled with 3.5 l of tap water. Plastic containers belonging to the same group were placed in the same artificial climate chamber (BIC-250, China), and each container represented one parallel replicate. During acclimation, the photoperiod was maintained at 12L : 12D, and the larvae were overfed daily with commercial red worms. The water was replaced with fresh water daily before each feeding.

### (b) Measurement of growth performance and food intake

For the 2019 acclimation, the total daily food intake (g d^–1^) of the 20 larvae from the same container was recorded every few days by calculating the difference between the initial and remnant food mass (wet mass) (electronic supplementary material, table S1). Thus, three values were obtained for each group at each timepoint. The body weight (g), length (cm) and body condition scores were recorded for each individual every few days (electronic supplementary material, table S2), but the average values for the larvae from the same container were calculated at each point (to avoid a pseudo-replication). Thus, three values were obtained for each group at each time point, and these data were used for statistical analysis and the calculation of the growth rate. The growth rate (g d^–1^) is defined as the daily average increment in body weight, and the food conversion rate was calculated as the ratio of growth rate to daily food intake. The body condition factor was calculated for each individual using the equation $(100\times \mathrm{body}\;\mathrm{weight})\;/\mathrm{body}\;\mathrm{lengt}h^{3}$.

### (c) Measurement of thermal preference

The thermal preference was tested for larvae acclimated in 2018. On days 30–45 after acclimation, the thermal preference of the acclimated larvae was determined in a thermal gradient ranging from 3 to 45°C. We followed the method of Hutchison and Hill [42] with some modifications. A detailed description and photograph of the experimental system are provided in electronic supplementary material, data S1 and figures S2 and S3. For each round of the experiment, one individual was initially placed in the centre of the thermal gradient (approximately 20–22°C) and allowed to swim freely. The larvae had 30 min to habituate to the gradient before measurement. The temperature of the water at its head position was recorded every 1 min for 30 min. As the larvae moved along the gradient during the observation periods, the temperatures at which the larvae stayed for the longest duration (greater than 4 min) were defined as the preferred temperature (PT) (see detailed explanation in electronic supplementary material, data S1). The PT of the larvae from different acclimation groups were measured alternately to avoid the batch effect. A total of 30 individuals were tested for their PT for each thermal group (electronic supplementary material, table S3). These larvae did not experience detrimental treatment, and thus they were transferred back to the acclimation course for subsequent transcriptional and metabolic analyses.

### (d) Transcriptional analyses

The larvae were collected on day 80 after acclimation (2018). After being weighed and euthanatized using MS-222, the larvae were dissected to collect the brain, heart, liver, gill, fore limb, dorsal skin and tail (1 cm from the cloaca site). The brain, heart, liver, gill and fore limb were weighed (electronic supplementary material, table S4) and all the samples were stored at −80°C.

#### (i) High-depth whole-length transcriptome

A high-depth transcriptome (greater than 40 GB) of pooled tissues was obtained using the third-generation sequence technology. In detail, the brain, heart, liver, gill, fore limb, dorsal skin and tail tissues of the larvae treated for 80 days were equally mixed. Total RNA was extracted and purified using Trizol (Invitrogen, Carlsbad, CA, USA) according to the manufacturer's instructions. After purification with poly-T oligo-attached magnetic beads, the cDNA was obtained using a SMARTer PCR cDNA Synthesis Kit. The SMRTbell library was constructed after amplification (KAPA HiFi PCR Kits) and screening (BluePippin). Sequencing was conducted on the PacBio platform. Polymerase reads with accuracy lower than 80% or shorter than 50 bp were removed. Circular consensus sequences were obtained after alignment, and the reads of insert (ROI) with accuracy lower than 80% or shorter than 50 bp were removed. The whole-length transcripts were obtained after clustering these ROIs with the isoform-level clustering algorithm. Subsequent analyses included open reading frame (ORF) prediction, LncRNA prediction and ORF function prediction (against NR and NT database) (see detailed information in electronic supplementary material, tables S5–S7 and electronic supplementary material, figure S4).

#### (ii) Comparative transcriptome analyses

High-depth transcriptomes (greater than 20 GB for each sample) of the single tissues (liver, heart, brain, fore limb, tail, dorsal skin and gill) were obtained using the second-generation sequence technology. In detail, the total RNA of each sample (*n* = 3 per tissue per group) was extracted and purified using Trizol (Invitrogen, Carlsbad, CA, USA). The library preparations (detailed in electronic supplementary material, data S1) were sequenced on an Illumina HiSeq 2500 platform (PE150 strategy) by Annoroad Gene Technology (Beijing). More than 20 GB of sequence data were obtained for each sample. The reads were annotated and quantified by querying against our whole-length transcriptome using RSEM and Bowtie2 (see the gene expression table in electronic supplementary material, table S8). Differentially expressed genes (DEGs) were identified using Student's *t*-test and Benjamini and Hochberg's (BH) correction at a threshold of *p* < 0.05 or *q* < 0.05. Functional enrichment analyses were conducted by querying the DEGs against the KEGG database (KOBAS 3.0, with default parameters).

### (e) Untargeted metabolomics

The liver, heart, brain, fore limb and tail tissues of the acclimated larvae were measured for metabolic profiles (*n* = 6 per tissue per group). For each sample, 100 mg tissue powder was ground in liquid nitrogen and extracted with 1 ml methanol : acetonitrile : water = 2 : 2 : 1 (v/v) (ultrasonication for 30 min × 2 and incubation at −20°C for 1 h). After centrifugation at 12 000 for 15 min (4°C), the supernatants were transferred into new tubes and freeze-dried. The samples were dissolved in 100 µl acetonitrile: water (1 : 1, v/v) and analysed using liquid chromatography (Agilent 1290 Infinity LC, USA) coupled with the quadrupole-time-of-flight mass spectrometry (Triple TOF 5600+, AB SCIEX, USA). Chromatographic analyses were conducted by Shanghai Bioprofile Technology Company Ltd (China). The parameters of chromatography and mass spectrometry were detailed in electronic supplementary material, data S1. The data were processed using XCMS with default parameters (http://metlin.scripps.edu/download/). The metabolites were identified by querying a standard library MS/MS spectrum built by Shanghai Bioprofile Technology Company Ltd. The relative abundances/concentrations of the metabolites are presented as the ion intensities of their molecular ion peaks (see the metabolite abundance table in electronic supplementary material, table S9). Differently expressed metabolites were screened using the Student's *t*-test and BH correction at a threshold of *p* < 0.05, or *q* < 0.05.

### (f) Statistical analysis

All the data were analysed using SPSS v. 25.0 (SPSS Inc., Chicago, USA). The Kolmogorov–Smirnov test was used to check whether the data deviated significantly from a normal distribution. Variations of PT between groups were analysed using the Student's *t*-test. The variations in body weight, length and food intake were analysed using the linear mixed model (repeated measurement) with temperature and timepoint as factors, and the interaction was considered. The variations in body condition factor and food conversion rate were also analysed using the linear mixed model, with body weight as a covariate. As larvae from the same container were unmarked and measured for body traits repeatedly, the average values were calculated for each container at each time point for statistical analyses to avoid a pseudo-replicate. Each container represented a replicate (*n* = 3 per group). The differences in organ weights between groups were analysed using ANCOVA, with body weight as a covariate. The differences in metabolome and transcriptome between groups were analysed using principal coordinates analysis and permutational multivariate analysis of variance. More detailed information is provided in electronic supplementary material, data S1. Graphs were generated using GraphPad prism 5 or ggplot2, an R package [43].

## 3. Results

### (a) Thermal physiology of *A. davidianus*

**Figure 1.**
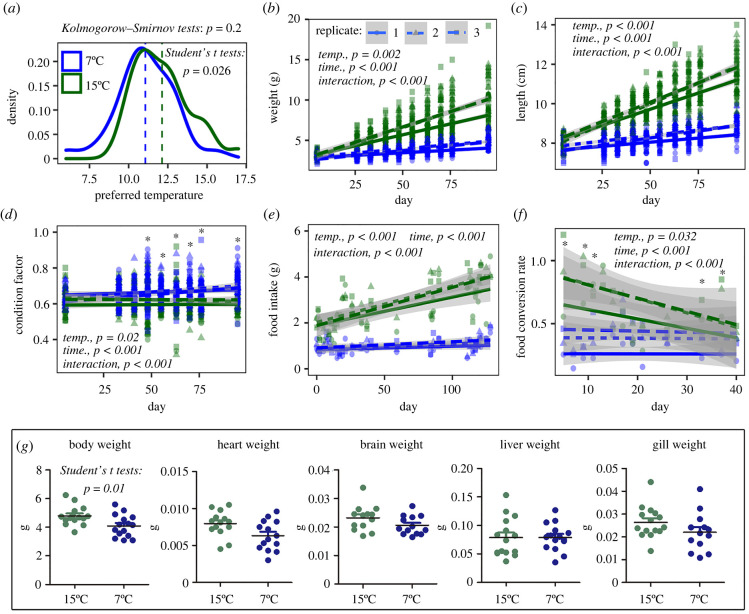
Thermal physiology of *A. davidianus* larvae. (*a*) Thermal preference of *A. davidianus* larvae acclimated for 30–45 days (*n* = 30 for each temperature). (*b*,*c*) Growth rate. Each point presents the body weight or body length of a single individual. Different line types and point shapes denote the parallel replicates (*n* = 3 per group). (*d*) Variations in body condition factors. (*e*) Daily food intake. (*f*) Food conversion rate. (*g*) Body and organ weight at the day 80 after acclimation (2018). The organ weights were analysed using ANCOVA, with body weight as a covariate (*p* > 0.05 for the interactive effects of temperature and body weight). The results suggested no significant effects of acclimation temperature and body weight on the organ weights at a threshold of *p* < 0.05. Asterisks denote significant difference (*p* < 0.05, simple effect analysis) between groups at the time point of measurement. (Online version in colour.)

The PT of *A. davidianus* fitted the normal distribution, with the average values ranging from 11 to 12°C. The PT of the cold-acclimated group was slightly lower (approx. 1°C) than that of the warm-acclimated group (*p* = 0.026; figure 1*a*). The cold-acclimated larvae showed a prominent lower growth rate in body size (temperature × time, *p* < 0.001 for body weight and length) than the warm-acclimated group. The body condition score did not vary with acclimation duration at 15°C, while it increased with time at 7°C, resulting higher body condition scores in the cold-acclimated larvae after 48 days of acclimation (*p* < 0.05, simple effect analysis) (figure 1*b*–*d*). The cold-acclimated larvae indicated decreased food intake (*p* < 0.001) and conversion rate (*p* = 0.032) in comparison to the warm-acclimated larvae (figure 1*e*,*f*). There were no significant differences in the heart, brain, liver and gill weights between the groups (*p* < 0.05; figure 1*g*).

### (b) Heterogeneity in cold responses between organs

**Figure 2.**
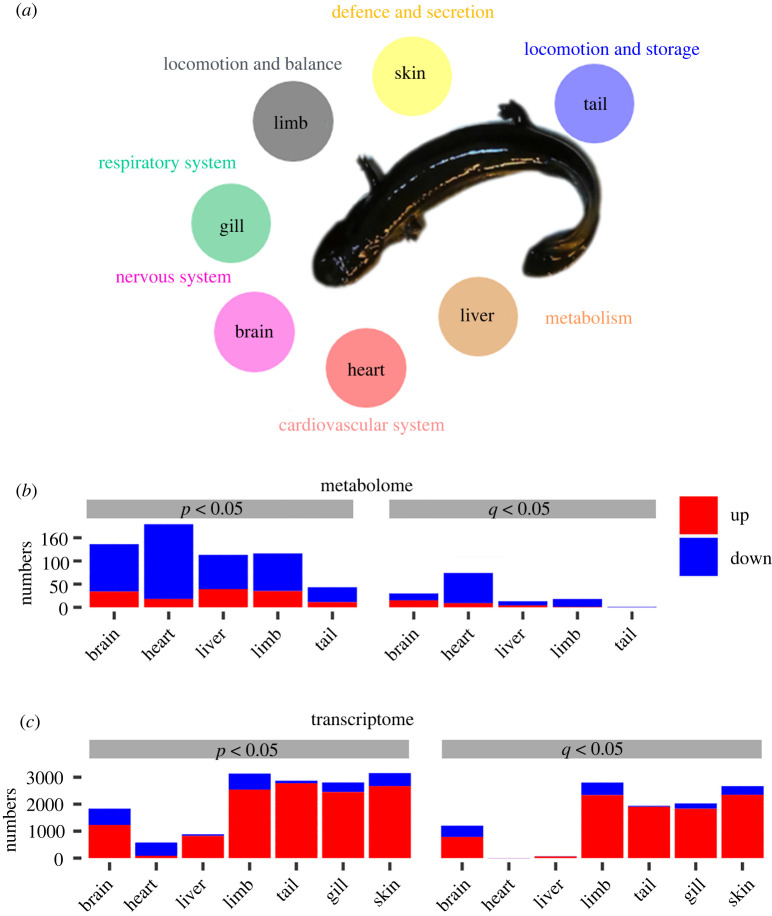
Comparative metabolomic and transcriptomic analyses. (*a*) Biological functions of the organs. (*b*,*c*) Numbers of differently expressed metabolites (*b*) or genes (*c*) between treatment groups (*p* < 0.05 or *q* < 0.05, Student's *t*-test and BH correction). The red colour indicates the proportion of upregulated items after cold treatment, and the blue colour denotes the downregulation.

The metabolic and transcriptional changes were analysed after cold acclimation (figure 2*a* and electronic supplementary material, figure S5). At the metabolic level, there were more downregulated metabolites than upregulated metabolites in all the studied organs, and the heart showed the most prominent metabolic variations (figure 2*b* and electronic supplementary material, figure S6*a*,*b*). At the transcriptional level, however, the numbers of the upregulated genes were greater than those of the downregulated genes in all the organs, except for the heart; and the heart showed the least transcriptional variation (figure 2*c* and electronic supplementary material, figure S6*c*,*d*).

**Figure 3.**
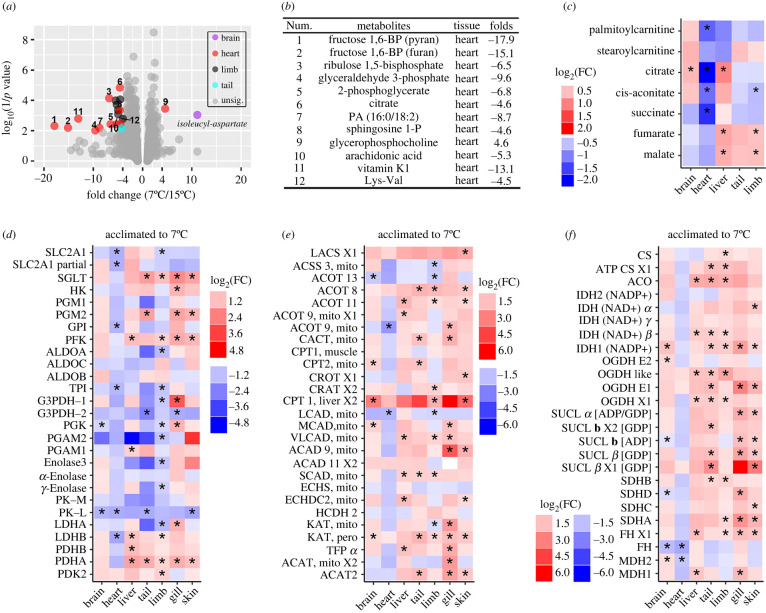
Metabolic reorganization for cold adaptation. (*a*,*b*) The metabolites changed drastically (fold change > 4, *p* < 0.01) under cold conditions. Note the large fold changes in glycolytic intermediates. (*c*) Variations in fatty acid beta-oxidation and TCA cycle intermediates. (*d*–*f*) The heat maps present the transcriptional changes in genes involved in carbohydrate catabolism (*d*), fatty acid catabolism (*e*) and the TCA cycle (*f*). *, *p* < 0.05. Detailed information on the gene abbreviations is provided in electronic supplementary material, table S10. (Online version in colour.)

More metabolites varied drastically (fold change > 4 and *p* < 0.01) to cold in the heart than in the other organs (figure 3*a*). In the heart, half of these metabolites were involved in glycol metabolism, e.g. fructose 1,6-diphosphate and glyceraldehyde 3-phosphate, which showed uniform downregulation under cold conditions (from 6.5- to 18-fold decrements; figure 3*b* and electronic supplementary material, figure S7). Additionally, several metabolites involved in fatty acid catabolism (e.g. palmitoylcarnitine) and the TCA cycle (e.g. citrate and succinate) also decreased in the heart of the cold-acclimated larvae, while their levels were either not changed or even increased in the others organs (figure 3*c*). At the transcriptional level, there were six differently expressed glycol-metabolic genes (e.g. phospho-glucose isomerase/GPI and triosephosphate isomerase/TPI; *p* *<* 0.05) in the heart, and all of them showed downregulation under cold conditions (figure 3*d*). The brain, liver, tail, limb, gill and skin showed 2 (up:down = 0:2), 5 (up:down = 5:0), 5 (up:down = 3:2), 14 (up:down = 5:9), 9 (up:down = 8:1) and 5 (up:down = 4:1) differently expressed glycol-metabolic genes. The heart also showed decreased transcription of genes involved in beta-oxidation and the TCA cycle, whereas the other organs showed prominent transcriptional upregulation of these two metabolic pathways (figure 3*e*,*f*).

### (c) Heterogeneity in cold responses between cellular processes

**Figure 4.**
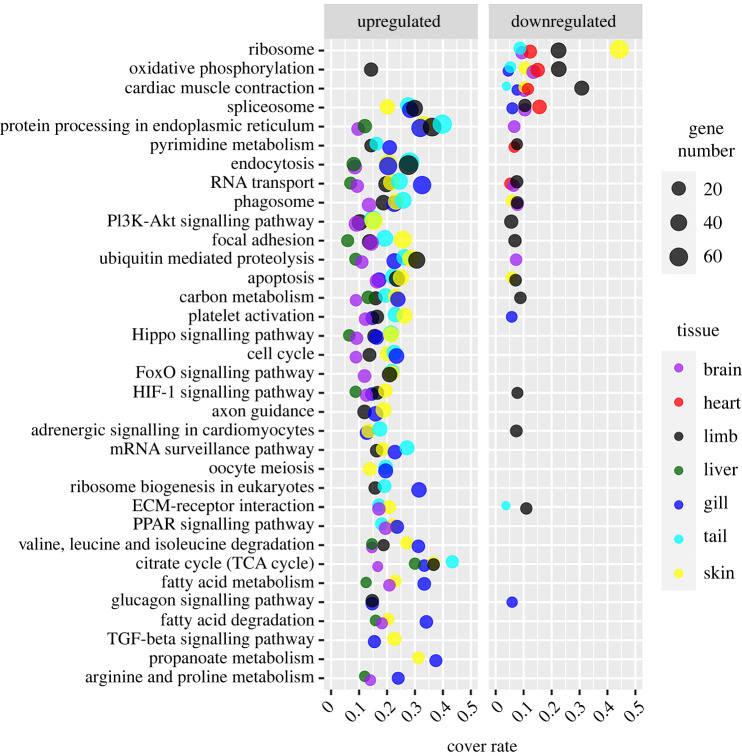
Metabolism-related pathways enriched by DEGs (*p* < 0.05). The enriched items (pathways) were screened at the threshold of *q* < 0.01 at least in one organ (KOBAS 3.0). For each item, its cover rate denotes the ratio of enriched gene number to the total gene number of the pathway. It should be noted that the enrichment of cardiac muscle contraction was also due to the DEGs of oxidative phosphorylation. (Online version in colour.)

The DEGs of each organ were queried against the KEGG database to highlight the cold-induced cellular processes. Most metabolism-related processes had the majority of their DEGs upregulated under cold conditions in the limb, tail, skin, gill, brain and liver (figure 4 and electronic supplementary material, table S11). However, there were two pathways, oxidative phosphorylation (e.g. a subunit of NADH dehydrogenase, cytochrome c reductase, cytochrome c oxidase and F-type ATP enzymes) and the ribosome components, which had the majority of their DEGs downregulated in the cold-acclimated group in almost all the organs including the heart. It should be pointed out that the HIF-1 signaling pathway was upregulated in the cold-acclimated larvae.

## 4. Discussion

We previously demonstrated cold acclimation in *A. davidianus* larvae, which was characterized by improved metabolic rate and locomotive performance under cold conditions [18]. In this study, we observed a comprehensive transcriptional upregulation of substrate metabolism (e.g. glycolysis, beta-oxidation and TCA cycle; figure 3*d*–*f*) and many other cellular processes (figure 4) in the organs, including liver, limb, tail, gill, skin and brain. This transcriptional compensation likely constitutes a molecular basis for the cold-induced physiological plasticity [44]. Transcriptional regulation of metabolic processes is commonly observed in cold acclimation processes [45,46]. Previous studies suggest that the mitochondrion is responsible for metabolic compensation [20,47]. Many mitochondrial components were transcriptionally upregulated in the fast muscle of the cold-acclimated zebrafish [46]. This is partly consistent with our results, as mitochondria are the site for beta-oxidation and the TCA cycle. However, not all the pathways of energy metabolism shared similar transcriptional variations. While the substrate catabolism was transcriptionally compensated, the components of oxidative phosphorylation, the downstream pathway, were downregulated (figure 4). Thus, we may expect decreased and increased maximum activities in oxidative phosphorylation and substrate catabolism, respectively, after cold acclimation (if measured at the same ambient temperature). The cold-acclimated larvae exhibit a higher respiration rate than their 15°C counterparts, which suggests that the maximum activity of oxidative phosphorylation is more than enough to deal with the metabolic flux from the substrate metabolism in both the control and cold-acclimated *A. davidianus* within the temperature scope of 7–15°C. Accordingly, the activity of substrate metabolism is likely the weak link, the ‘floor’, for the capacity of energy metabolism in *A. davidianus* larvae, which undergoes compensation. In turn, the transcriptional downregulation of oxidative phosphorylation may set a dropping ‘ceiling’ for thermal compensation in these organs. The heterogeneity in cold acclimation between cellular processes may be involved in the shift in cold adaptive strategy from compensation to depression with the drop in temperature at the organ level.

Cold acclimation heterogeneity is also observed at the higher organizational level. Our results indicate prominent heterogeneity in cold acclimation between the organs. As we discussed above, the liver, limb, tail, gill, skin and brain exhibit comprehensive transcriptional upregulation of the genes involved in numerous cellular processes (figures 2*c* and 4), and these organs suffered less dramatic changes in metabolome after cold-acclimation (figure 2*b*). Most importantly, their levels of intermediates in energy metabolism were maintained or even increased after acclimation. Thus, we may expect an increase in the maximum metabolic capacity of these organs in comparison to their 15°C counterparts if oxygen and nutrient are sufficient. The brain, liver, tail and limb accounts for a large proportion of the individual metabolic rate and are responsible for the maintenance of locomotive activities. Their cold-induced changes can explain the increased metabolic rate and lower critical temperature in the cold-acclimated *A. davidianus* larvae [18]. Interestingly, we observed increased cold preference in the cold-acclimated larvae (figure 1*a*). In *Drosophila melanogaster*, increased energy metabolism is responsible for the cold preference and chill tolerance of the DmDG-mutated strain, as staying in a cooler environment can reduce their energy production and thus benefit their energy homeostasis [48]. It may also explain the observation in *A. davidianus* larvae.

**Figure 5.**
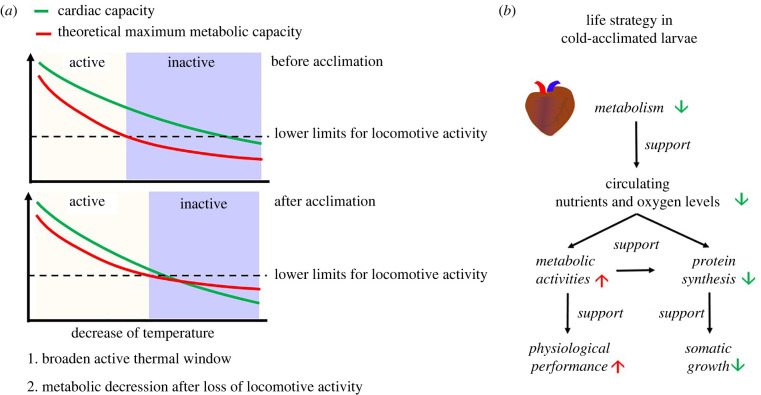
Deduced cold adaptive strategy in *A. davidianus* larvae. (*a*) Organ heterogeneity in cold acclimation likely directed the shift in cold adaptive strategy from compensation to depression with the drop in temperature. (*b*) Life strategy in cold-acclimated larvae at 7°C. (Online version in colour.)

However, the metabolic capacity of tissues is not only determined by the enzyme levels and theoretical maximum activities but also affected by the availability of circulating nutrients and oxygen [49]. The metabolome of the heart is more sensitive to cold than other organs, with weak transcriptional compensation (figure 2*b*,*c*), and its catabolism of the three major substrates (carbohydrate, lipid and amino acid) is even downregulated at the transcriptional level. Combining the passive thermodynamic effect and active regulation of heart energy metabolism, we can expect a more dramatic decrease in the cardiac function of *A. davidianus* larvae under cold conditions than other physiological functions. This is supported by the decreased level of intermediates of energy metabolism in the heart (figure 3*a*). In combination with the upregulation of the HIF signalling pathway, these results suggest that the heart is likely the organ system that initially limits the cold performance of *A. davidianus* larvae. It supports the oxygen- and capacity-limited thermal tolerance hypothesis [50,51]. Given that cardiac function is fundamental to the delivery of oxygen and nutrient to other tissues, its thermal property likely constitutes a systemic ‘ceiling’ for metabolic capacity and compensation intensity at the whole-organism level. Although this ‘ceiling’ did not constrain the basal metabolism at 7°C [18], it may become a compelling limiting factor for metabolism at cooler temperatures. Reduced heart rate is a major feature of mammal hibernation, and adaptive changes in gene expression in the heart before hibernation may be indispensable for acquiring cold resistance [52]. Therefore, the cold-acclimation heterogeneity between organs may have orchestrated the shift in cold adaptive strategy from compensation to depression with the drop in temperature (figure 5*a*).

Cold acclimation likely modified the resource allocation strategy of the cold-acclimated *A. davidianus* larvae. The cold-acclimated larvae showed a decreased food conversion rate. This can be attributed to two potential reasons, decreased assimilation rate under cold conditions and reorganization in resource allocation between body mass accumulation (including storage and somatic growth) and metabolic maintenance. The transcriptional downregulation of the ribosomal component in the cold-acclimated larvae (figure 4) validates the presence of the latter, as ribosome biogenesis is a major regulation target of protein synthesis and growth [53]. Moreover, while the somatic growth of the cold-acclimated larvae decreased dramatically, their body condition index increased. Given that body condition is commonly considered as the quantity of nutrient reserves, determining the fitness and survival capacity of individuals [54], it is likely that the cold-acclimated larvae invested more resources into preservation rather than growth in body size [55,56]. Macromolecule synthesis during somatic growth is the most resource and energy-consuming cellular process [57], and it is susceptible to a reduction in circulating nutrient levels and oxygen availability [58,59]. Thus, decreased cardiac function could, at least partly, explain the suppression of protein synthesis and somatic growth in the cold-acclimated larvae. In combination with the compensation in metabolism in the other organs, organ heterogeneity in cold acclimation likely directs a systemic reorganization in resource allocation, characterized by downregulation of somatic growth while compensating for physiological activities and body conditions (figure 5*b*). Such an adjustment should be beneficial to their maintenance and survival under cold conditions. This is especially true for *A. davidianus*, the ambush foragers, who may not always have the opportunity for food intake, and likely rely on their reserve to maintain life activities at most times in the wild. Although thermal compensation can improve their physiological performance and thus forage opportunity under cold conditions, the resource acquisition may not keep the pace of consumption if maintaining similar compensation for maintenance and somatic growth. Thus, organ heterogeneity in thermal acclimation is a significant mechanism in regulating the thermal adaptation of *A. davidianus* larvae.

## 5. Conclusion

In this study, we investigated the transcriptional and metabolic variations underlying the cold acclimation of *A. davidianus* larvae. These data showed evidence of thermal compensation at the transcriptional level, and indicated prominent heterogeneity in cold acclimation at the organ and molecular levels. While most organs showed transcriptional upregulation of metabolic activities, the heart exhibited a downregulation, with their levels of intermediates in energy metabolism decreasing dramatically. Consequently, although the physiological and metabolic activities were compensated at the organ level, cardiac function might constitute a ‘ceiling’ to constrain the space for metabolic compensation, especially under cooler conditions. We thus may expect a shift in cold adaptive strategy from compensation to depression with the drop in temperature. Overall, organ heterogeneity in cold acclimation likely shapes the metabolic strategy to maximize overall fitness.

## Data Availability

The database used for the analyses presented here are available from the electronic supplementary material [60]. Sequencing data and relevant files have been uploaded to https://ngdc.cncb.ac.cn/gsa/, with the accession number CRA006550 [61].
